# The Swiss Perimenopause Study – study protocol of a longitudinal prospective study in perimenopausal women

**DOI:** 10.1186/s40695-020-00052-1

**Published:** 2020-07-20

**Authors:** Jasmine Willi, Hannah Süss, Ulrike Ehlert

**Affiliations:** 1 Clinical Psychology and Psychotherapy, University of Zurich, Binzmühlestrasse 14, 8050 Zurich, Switzerland; 2 URPP Dynamics of Healthy Aging Research Priority Program, University of Zurich, Zurich, Switzerland

**Keywords:** Perimenopause, Menopausal transition, Reproductive aging, Women’s health, Resilience, Depression, Depressed mood

## Abstract

**Background:**

The perimenopause is associated with considerable biopsychosocial changes. The majority of women manage to adjust to these changes and cope well with the shift from reproductive to non-reproductive life. However, some women develop burdensome physical and psychological symptoms during the perimenopause. A strong link between menopausal complaints and depressed mood has been shown in this regard. To date, the decisive factors determining whether a woman will successfully achieve a healthy transition remain unclear. Thus, the purpose of this study is to investigate a range of theory-based markers related to health in perimenopausal women.

**Methods:**

The Swiss Perimenopause Study comprises a sample of 135 healthy perimenopausal women aged 40–56. A variety of health-related genetic, epigenetic, endocrinological, physiological, and psychosocial markers associated with the menopausal transition are investigated over a period of 13 months.

**Discussion:**

The Swiss Perimenopause Study will contribute to a better understanding of the biopsychosocial processes associated with the perimenopause, which should help to improve the clinical care of women undergoing the menopausal transition.

## Background

The perimenopause is related to substantial biological, psychological and social changes [1–3]. It describes the biological shift from reproductive to non-reproductive life, which is associated with significant alterations in the female hormonal system. The perimenopause is considered as a phase of strong fluctuations in sex hormones such as estradiol and progesterone [4, 5]. Endocrinological variations have been correlated with a higher risk of burdensome physical and psychological symptoms [6–12], subsumed under the term “menopausal complaints”. These complaints are generally divided into psychological, somato-vegetative, and urogenital symptoms. However, in spite of the major biopsychosocial changes and challenges, most women adjust well to the perimenopause, reporting a good quality of life and an overall positive well-being [13]. It is assumed that resilience might be a key factor in determining whether or not a woman will be negatively affected by menopausal symptoms [14]. Women with high levels of resilience are believed to more effectively and successfully adapt to the substantial changes in the perimenopause [15, 16] compared to less resilient women. In turn, women with lower levels of resilience are suggested to be more affected by menopausal complaints like vasomotor symptoms or sleep disturbances. Such symptoms may then co-occur and have been shown to interact with depressed mood [17–19], suggesting the perimenopause as a window of vulnerability for mood disturbances [20]. Indeed, prevalence rates of depressed mood during the perimenopause are particularly high [21]. Consequently, the investigation of depression in the course of the menopausal transition has gained importance over the last decades.

The attention paid to women’s health research has gradually increased since the 1970s [22]. One of the first longitudinal studies on the menopausal transition was the Massachusetts Women’s Health Study (MWHS) [23], followed by the Seattle Midlife Women’s Health Study (SMWHS), which collected data over 23 years [24]. In Norway, a large cohort study, the Nord-Trøndelag Health Study (HUNT) [25], began in 1984 and is considered one of the largest health studies ever performed. Further large studies on the menopausal transition, such as the Study of Women’s Health Across the Nation (SWAN) [26], the Penn Ovarian Aging Study (POAS) [4], or the Harvard Study of Moods and Cycles [27], followed in the US. Extensive research on women in midlife has also been conducted in Australia, within the Australian Longitudinal Study on Women’s Health (ALSWH) [28] or the Melbourne Women’s Midlife Health Project (MWMHP) [29]. Depressed mood was one of the focal points across these studies, and all of them highlighted the increased risk for new or recurrent depressed mood, emphasizing the prevalence peak during the menopausal transition. This previous research formed an important basis for the understanding of depressed mood in middle-aged women and the associated changes and challenges.

Previous studies frequently included women across all three menopausal stages, i.e. the pre-, the peri-, and the postmenopause. Such a selection procedure seems to be highly expedient in terms of identifying critical time points in the development of symptoms in the progression from reproductive to non-reproductive life. Moreover, this sample selection also provides the opportunity to compare the participants across the different menopausal stages. However, in terms of investigating the perimenopause as a critical window of increased biopsychosocial changes and associated complaints, and in order to specify the involved processes, it is essential to focus specifically on the perimenopausal phase.

Therefore, the Swiss Perimenopause Study employs a longitudinal design examining women in the perimenopause only and assessing a variety of genetic, epigenetic, endocrinological, physiological and psychosocial factors. As such, we aim to investigate psychosocial factors like personality traits, self-esteem, self-compassion, perceived stress, emotion regulation and coping, or social support. Furthermore, physiological parameters like blood pressure and pulse, heart rate variability, hand grip force, or body composition are assessed as measures associated with healthy aging. Body weight and body height are captured in order to take into account participants’ body mass index (BMI) when investigating biomarkers. Additionally, we measure the length of the index finger and the ring finger in order to calculate the second to fourth digit ratio (2D:4D). Recent research showed that a higher 2D:4D is associated with a higher age at menopause, while a lower 2D:4D (interpreted as suggesting a higher androgen exposure during the prenatal phase) is associated with a lower age at menopause [30]. As our workgroup has shown that looking younger than one’s actual age can be related to a variety of health outcomes [31], we take standardized facial photographs of all participants. The full list of biopsychosocial factors included in this study is shown in Tables 1 and 2. The comprehensive investigation of possible predictors of physical and psychological complaints appears particularly promising regarding the detection of underlying processes such as epigenetic variations or specific hormonal patterns.

**Table 1:** Psychosocial measures included in the Swiss Perimenopause Study

Ref.	Construct	Assessment instrument	Authors	Items
[32]	Body image	Body Image Questionnaire (Fragebogen zum Körperbild; FKB-20)	Clement & Löwe, 1996	20
[33]	Chronic stress	Trier Inventory for Chronic Stress, short version (TICS-K)	Schulz et al., 2004	30
[34]	General health	General Health Questionnaire (GHQ-12)	Schmitz et al., 1999	12
[35]	Coping	COPE Inventory, short version	Knoll et al., 2005	28
[36]	Depressive symptoms	German Version of the Center of Epidemiological Studies Depression Scale (CES-D; Allgemeine Depressionsskala; ADS-L)	Hautzinger & Bailer, 1993	20
[37]	Emotional intelligence	Emotional Intelligence Questionnaire, German version (TEIQue)	Freudenthaler et al., 2008	30
[38]	Emotion regulation	Emotion Regulation Questionnaire, German version (ERQ)	Abler & Kessler, 2009	10
[39]	Eating behavior	Eating Disorder Examination Questionnaire (EDE-Q)	Hilbert & Caffier, 2006	28
[40]	Explicit motives	Life Goal Questionnaire (GOALS)	Pöhlmann & Brunstein, 1997	24
[41]	Intrasexual competition	German version of the Scale for Intrasexual Competition (ICS)	Fiacco et al., 2018	12
[42]	Life events	German version of the Life Experience Survey (LES)	Pluess et al., 2012	57
[43]	Life satisfaction	Satisfaction with Life Scale (SWLS)	Glaesmer et al., 2011	5
[44]	Mastery	Pearlin Mastery Scale (PM)	Pearlin et al., 1981	7
[45]	Menopausal symptoms	Menopause Rating Scale (MRS II)	Hauser et al., 1999	11
[46]	Physical and mental symptoms	Brief Symptom Inventory (BSI-18)	Spitzer, 2011	18
[47]	Pessimism/Pessimism	Life Orientation Tests (LOT-R)	Glaesmer et al., 2008	10
[48]	Parental bonding	German version of the Parental Bonding Instrument (PBI; Fragebogen zur elterlichen Bindung; FEB)	Lutz et al., 1995	25
[49]	Personality	Short version of the Big Five Inventory (BFI-K)	Rammstedt & John, 2005	21
[50]	Perceived stress	German version of the Perceived Stress Scale (PSS-10)	Klein, 2016	10
[51]	Premenstrual symptoms	PMS Inventory	Ditzen et al., 2011	30
[52]	Promiscuity	Sociosexual Orientation Index (SOI-S)	Penke & Asendorpf, 2008	9
[53]	Relationship experiences	Experiences in Close Relationships (ECR-RD 12)	Ehrenthal, 2009	12
[54]	Relationship quality	German version of the Relationship Assessment Scale (RAS)	Sander & Böcker, 1993	7
[55]	Resilience	Resilience Scale 11 (RS-11)	Schumacher et al., 2005	11
[56]	Retrospectively assessed relationship experiences	Sequence data analysis	Abbott, 1995	10
[57]	Self-compassion	German Version of the Self-Compassion Scale (SCS-D)	Hupfeld & Ruffieux, 2011	26
[58]	Self-esteem	Rosenberg Self-esteem Scale (RSE)	von Collani & Herzberg, 2003	10
[59]	Sense of coherence	Revised Sense of Coherence Scale (SOC-R)	Bachem & Maercker, 2016	13
[60]	Sexual desire	German version of the Decreased Sexual Desire Screener (DSDS)	Clayton et al., 2009	5
[61]	Sexual function	Female Sexual Function Index (FSFI / FSFI-LL)	Berner et al., 2004	19
[62]	Sleep	Pittsburgh Sleep Quality Index (PSQI)	Backhaus, 2002	19
[63]	Social support	Berlin Social Support Scales (BSSS)	Schulz & Schwarzer, 2003	17
[64]	Spirituality	German version of the Expressions of Spirituality Inventory-Revised (ESI-R)	Proyer & Laub, 2017	32
[65]	Traumatic experiences	Childhood Trauma Scale Short Form (CTQ-SF)	Klinitzke et al., 2011	28

**Table 2:** Biological parameters included in the Swiss Perimenopause Study

Source	Label	Parameter
Saliva (SaliCaps)	Endocrine parameters	• Cortisol
		• Alpha amylase
		• Testosterone (T)
		• Dehydroepiandrosterone sulfate (DHEA-S)
		• Estradiol (E2)
		• Progesterone (P)
Blood (DBS)	Endocrine and inflammatory parameters	• C-reactive protein (CRP)
		• Interleukin-6 (IL-6)
		• Sex hormone-binding globulin (SHBG)
		• Luteinizing hormone (LH)
		• Follicle-stimulating hormone (FSH)
	Genetic and epigenetic parameters	• Estrogen receptor genes (ER1, ER2, GPER)
		• Glucocorticoid receptor (NR3C1)
		• Serotonin transporter (5-HTTLPR)
Blood pressure monitor	Peripheral physiological measures	• Blood pressure (BP)
		• Pulse
Ava bracelet	Peripheral physiological nighttime measures	• Skin temperature
		• Ambient temperature
		• Electrodermal activity (EDA)
		• Heart rate (HR)
		• Heart rate variability (HRV)
		• Blood circulation of the skin
		• Body acceleration
		• Breathing rate
		• Sleep phases
Body scale	Anthropometric measures	• Body weight
Scale		• Height
BIA		• Body composition
Vernier caliper		• Finger lengths
Hand dynamometer		• Grip force
Camera		• Standardized facial photograph

Data concerning genetic and epigenetic markers associated with the menopausal transition are still scarce. However, valuable information might be gained by analyzing gene-environment interactions and the effect of epigenetic modifications on the menopausal transitioning [66]. For this purpose, we aim to investigate the estrogen receptor genes (ER1, ER2, GPER), the glucocorticoid receptor (NR3C1) as well as the serotonin transporter (5-HTTLPR) and the respective relationships with a resilient menopausal transition or the development of depression in the perimenopause. For example, a recent study by our workgroup found a positive relationship between the methylation of the ERα enhancer and depressed mood in women around the menopause [67]. Consequently, one goal is to investigate the autoregulation between estradiol and the estrogen receptor genes with regard to methylation patterns and subsequent effects on the participants’ mood.

Endocrinological parameters are commonly assessed in studies on the menopausal transition. Previous research in this regard showed a significant relationship between sex steroids and menopausal complaints [68–70]. Several studies by our workgroup showed that cortisol or alpha amylase play a key role in the reactivity of physiological stress systems [71–73], and inflammatory markers associated with various psychopathologies change over the menopausal transition [74]. Therefore, their assessment is included in this study. To maximize the informative value and comparability of the forthcoming results, the Swiss Perimenopause Study is designed to conduct a close endocrinological monitoring of all participants, enabling progression analyses of individual hormone profiles. Standardized methods of analysis are used, as described in the methods section.

By repeatedly assessing physiological data during the night, it may be possible to address questions about the relationship of sleep disturbances and vasomotor symptoms in women suffering from hot flushes and night sweats. To the best of our knowledge, the Swiss Perimenopause Study represents the currently largest study investigating such a wide range of health-related biopsychosocial markers in perimenopausal women.

The purpose of the Swiss Perimenopause Study is to gain a deeper understanding of the biopsychosocial factors and processes associated with 1) resilience and health in the perimenopause, and 2) the development of new or recurrent depression. One key goal of this study is to investigate biopsychosocial markers, determinants, and processes related to a resilient menopausal transitioning. In this regard, we aim to examine psychosocial variables such as optimism, emotional stability, or self-esteem as well as biological markers such as female sex steroids and gonadotropins, stress hormones or inflammatory parameters and their relationship with well-being and health. We assume that women with higher psychosocial resilience (e.g. higher optimism, higher emotional stability, higher self-esteem) and higher levels of female sex steroids will be more successful in their adaptation to changes and challenges associated with the menopausal transition. A second key goal is to investigate the biopsychosocial determinants and associations of perimenopausal depression. To achieve this, we aim to investigate specific risk factors for depressive symptoms in the perimenopause and to examine the effect of endocrinological alterations and psychosocial changes and their effect on the women’s mood. We assume that a history of depression and fluctuations of sex steroids are major risk factors for perimenopausal depression. As such, it is our endeavor to expand and develop the existing knowledge on healthy menopausal transitioning. To our knowledge, this is the first study to investigate both depression and resilience within the perimenopause.

## Methods

### Design

**Fig. 1:**
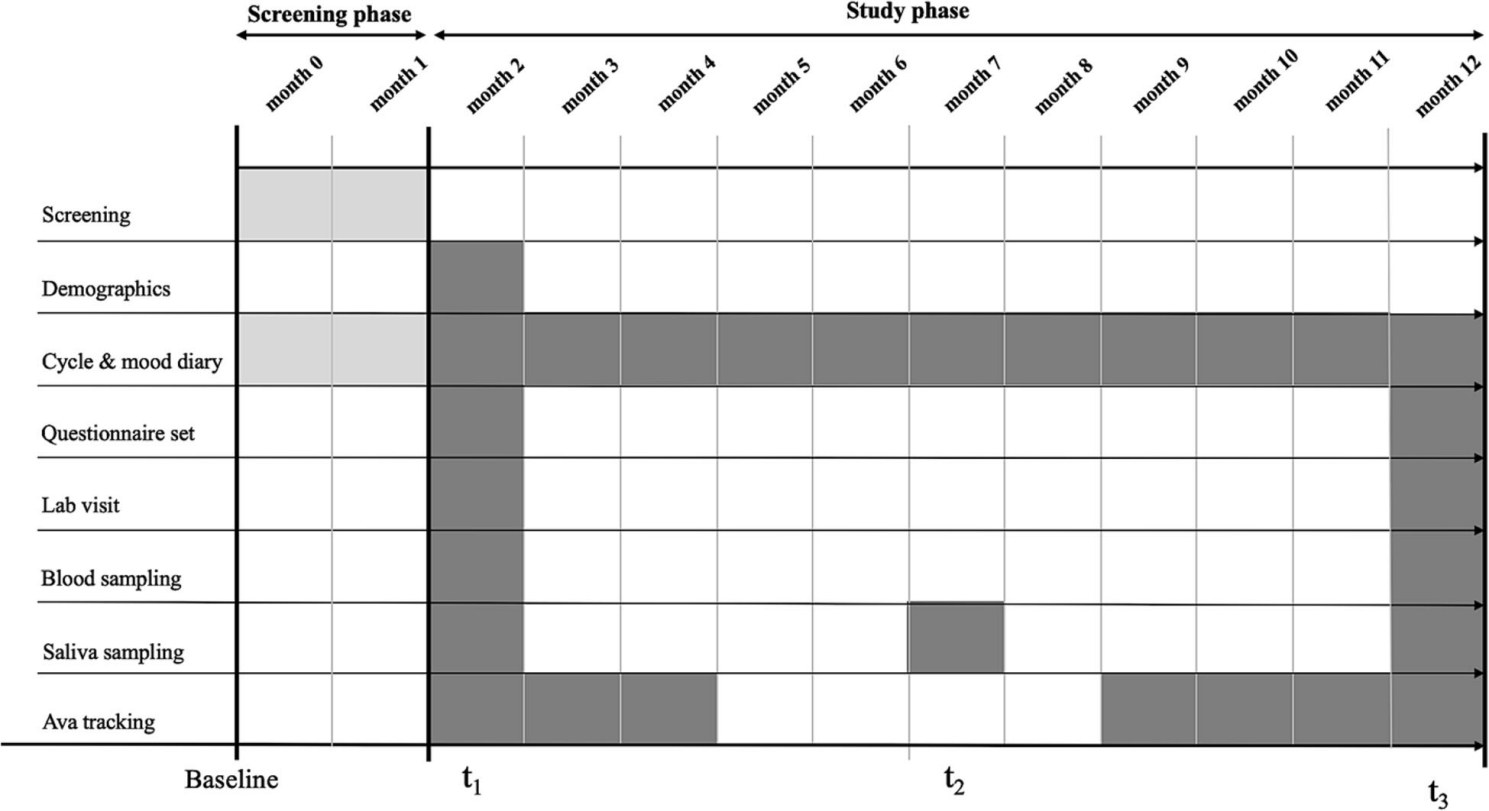
Project Design

The Swiss Perimenopause Study is an exploratory, longitudinal, single-center, national study examining different facets of female menopausal development over 13 months. Data are being collected between June 2018 and January 2021. The study is divided into a screening phase and a study phase. After the application of the strict inclusion criteria, interested women were screened for about 2 months for our study purposes. Subsequently, the actual study phase began, which will last for 11 months. Relevant genetic, epigenetic, endocrinological, physiological, and psychological aspects of the menopausal transition are investigated by means of different measurement methods. The participation includes two visits to our laboratory at the Institute of Psychology, University of Zurich. The standardized lab visits include blood sampling and the assessment of different peripheral physiological (blood pressure, pulse) and anthropometric parameters (body weight, body height, bioelectrical impedance analysis (BIA), second to fourth digit ratio, grip strength, standardized facial photograph). During the months between the two lab visits, participants independently collect saliva samples at home, measure various physiological parameters with the Ava bracelet (Ava AG, Zürich, Switzerland), complete a menstrual cycle and mood diary, and answer a set of different psychosocial questionnaires. An overview of the study design can be found in Fig. 1. To date, the screening phase and t_1_ have been completed.

### Sample

The population-based sample of 135 perimenopausal women aged 40–56 were recruited through mailing lists, social media, context-specific online forums, flyers as well as newspaper and magazine articles. For the sample size estimation of the planned project, we performed an a priori sample size estimation analysis using G*Power 3.1 for different statistical analyses such as correlation analysis, linear regression analysis, linear multiple regression analysis, or Wilcoxon signed-rank test with an α-level < .05 [75].

From June 2018 to December 2019, a total of 1121 women showed interest in participating in the Swiss Perimenopause Study by completing an online screening questionnaire. The eligibility for study participation was checked by following inclusion and exclusion criteria (Table 3).

**Table 3:** Inclusion and exclusion criteria

**Inclusion criteria**	
• Female sex	
• Age 40 to 60 years	
• Perimenopausal status	
• Good knowledge of the German language	
• Good to excellent self-reported health condition	
**Exclusion criteria**	
• Acute or chronic somatic disease	
• Acute or chronic mental disorder	
• Psychiatric drug use	
• Psychotropic drug use in the last 2 months	
• More than two standard units of alcoholic beverages a day	
• Pregnancy in the last 6 months	
• Current use of oral contraceptives or hormone therapy in the last 6 months	
• Postmenopausal status (no menstrual period in the last 12 months)	
• Premenopausal status (regular menstrual cycle)	

**Fig. 2:**
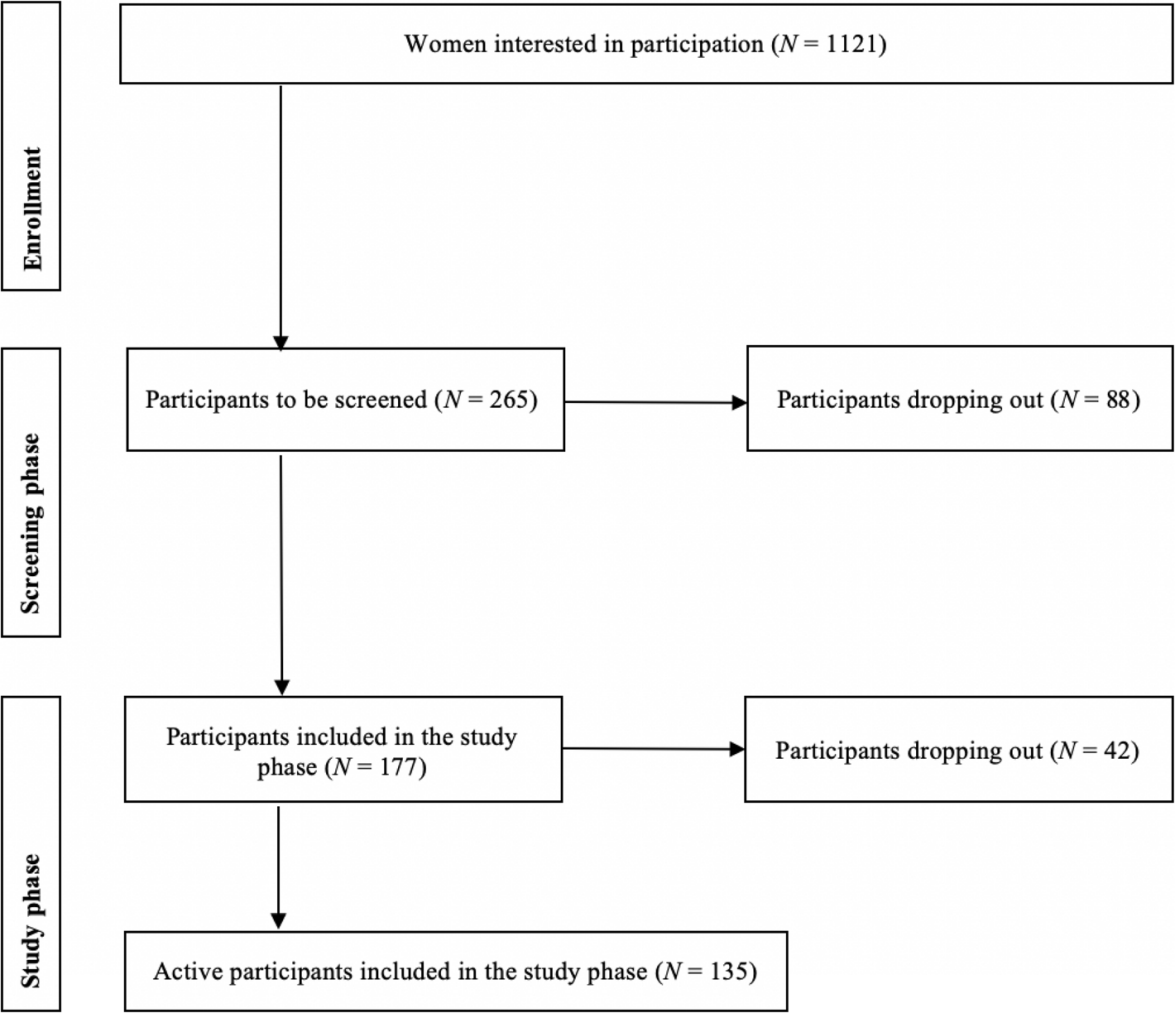
Sample inclusion and elimination process

Major reasons for exclusion included pre- or postmenopausal status (*n* = 717), poor subjective health (*n* = 97), age below 40 or above 60 years (*n* = 58), hormone therapy in the last 6 months (*n* = 34), acute or chronic somatic or mental illness (*n* = 22), and psychiatric or psychotropic drug use (*n* = 11). The sample inclusion and elimination process is displayed in Fig. 2. Participants eligible for the study were granted access to the online menstrual cycle and mood diary for the two-month screening phase. Within this phase, the perimenopausal status was ensured. The perimenopausal status was defined according to the Stages of Reproductive Aging Workshop *+ 10* (STRAW) criteria, representing the most frequently used classification system [76]:

1. Early menopausal transition: increased variability in menstrual cycle length, defined as a persistent difference of ≤7 days in the length of 10 consecutive cycles.
2. Late menopausal transition: the occurrence of amenorrhea of 60 days or longer.

In addition to the exclusion criteria, the screening questionnaire also recorded whether the currently subjectively healthy participants show a history of depression. In this regard, prior depression must either have been previously diagnosed by an expert, or as self-report, in accordance with the criteria for major depression according to the Diagnostic and Statistical Manual of Mental Disorders, Fifth Edition (DSM-5).

### Retention efforts

Numerous efforts have been undertaken to retain the eligible participants throughout the study. These include personal and consistent contact from the research staff, daily accessibility to the research staff, annual Christmas cards, reminders about the data collection by text message or telephone calls, and regular menopause-specific information on our homepage and social media profiles. Furthermore, participants were given an individual health profile after the evaluation phase and are given various incentives throughout the different time points during the study (e.g. vouchers for massage, wellness and spa treatments, free gym subscription, cosmetics, food vouchers).

### Data collection

During the entire screening and study phase, participants are asked to mark bleeding days in their individual paper-and-pencil cycle calendar. This provides an easily accessible method to remember the days of bleeding for the biweekly online mood and cycle diary. Every 2 weeks, participants are reminded to complete the online cycle and mood diary, which assesses specific cycle conditions, menopausal complaints, and mood disturbances over the last 2 weeks. At the beginning of the study phase (t_1_), further demographic data (e.g. marital status, occupation) and a variety of psychosocial factors were assessed via online questionnaires. Psychosocial measures include psychological traits, physical and mental health outcomes, behavioral aspects, and interpersonal factors. The validated self-report questionnaires assessed in the course of the study can be found in Table 1, which lists the measured constructs, the name of the assessment instrument, the authors, and the number of items of the instrument (Table 1). The questionnaires are completed at the beginning and at the end of the study (t_1_ and t_3_). Participants receive the respective links by e-mail. All questionnaires need to be completed consecutively.

Two standardized laboratory visits to the Institute of Psychology, University of Zurich are planned within the study phase, at t_1_ and t3. Women are instructed a) not to drink caffeinated drinks (e.g. coffee, black or green tea, energy drinks) or alcohol for at least 48 h (2 days) prior to the visit, b) not to engage in intensive physical activity for at least 24 h (1 day) prior to the visit, and c) not to eat, drink or engage in physical effort (e.g., cycling) on the morning of the examination. The laboratory sessions are standardized. Participants arrive at the laboratory at 7:45 am. At the first laboratory visit (t_1_), a well-instructed psychologist first conducted the German Version of the Structured Clinical Interview for DSM-IV [77] to ensure that all the participants were mentally healthy at the beginning of the study. Otherwise, participants were excluded.

At both laboratory visits (t_1_ and t_3_), small capillary blood samples are drawn from the participant’s finger. Blood pressure and pulse (Medisana MTV upper arm blood pressure instrument) are measured and body height and weight are assessed. In addition, the body composition is determined using bioelectrical impedance analysis (BIA, Biocorpus RX4000, Idiag AG, Fehraltdorf, Switzerland). This instrument is a fully digital, phase-sensitive, four-channel impedance-measuring device. Each channel applies a 50 kHz alternating current to measure resistance, reactance and phase angle. To measure the second to fourth digit ratio (2D:4D), the length of the index finger and the ring finger is measured (Digital Caliper Micrometer Vernier Gauge Tool). Accordingly, the grip strength is assessed (Baseline Hydraulic Hand Dynamometer). The laboratory sessions also include a standardized facial photograph. These photos are used for self-rated attractiveness as well as externally rated attractiveness. For the latter, independent and external examiners (*N* = 10) rate the standardized facial photographs of the participants according to the perceived age, attractiveness and health on a scale from 0 to 100. The assessments are conducted via presentation of the photographs on a computer screen. The examiners have signed a binding declaration of confidentiality.

Within all laboratory sessions, the investigator also asks a short, standardized set of questions about factors and activities potentially biasing the current hormone concentrations. Subjects are instructed to autonomously collect saliva samples in a non-invasive manner with saliva sampling tubes (SaliCaps, IBL International GmbH, Hamburg, Germany). Furthermore, the participants receive written instructions for each step of the saliva sampling. The participants have been provided with 100 SaliCaps (IBL International GmbH, Hamburg, Germany) to collect one daily morning saliva sample for 3 months in total (i.e. 28 daily samples per month during t_1_, t_2_ and t_3_) under standardized conditions. To investigate the cortisol awakening response (CAR) as well as fluctuations throughout the day, four additional saliva samples are collected on the first 2 days of the month of t_2_ and t_3_. The additional saliva samples are timed 30 and 60 min after the first saliva sample, at 12:00 pm and before going to bed. The exact sampling times as well as specific information on caffeine or alcohol consumption, sports or current gum bleeding are recorded in a protocol. Participants are reminded to start the saliva sampling at the beginning of t_1,_ t_2_, and t_3_.

During the first lab visit, the participants were also introduced to the Ava bracelet (Ava AG, Zürich, Switzerland), which they were asked to wear at night for a total of 6 months. The Ava bracelet assesses raw data of nine different nighttime peripheral physiological parameters: skin temperature, ambient temperature, electrodermal activity, heart rate, heart rate variability, blood circulation of the skin, body acceleration, breathing rate, and sleep phases.

An overview of all biological parameters assessed in the course of the study can be found in Table 2, which lists the sources of the parameters, the label, and the name of the specific parameter (Table 2).

### Data handling

A link for the online screening was provided on the study homepage. Women interested in participation were led to the online platform Unipark, where they were asked to provide initial, brief information followed by a declaration of consent. All questionnaires at baseline, t_1_ and t_3_ (www.unipark.com), the data analysis of retrospectively assessed relationship experiences (www.qualtrics.com), as well as the mood and cycle diary (www.findmind.ch), are completed online. The participants were explicitly informed about the study design, the aims, the procedure and the expected duration of each part of the assessment. Data security is ensured at all times. All calendars and protocols are paper-and-pencil-based. The online platform Unipark is reliably protected from any external access. The BSI-certified (Bundesamt für Sicherheit in der Informationstechnik [Federal Office for Information Security]) data center is subject to requirements of high data protection and safety in conformity with ISO (International Organization for Standardization) 27,001 based on the IT basic protection. Qualtrics is hosted by trusted data centers that are independently audited using the industry standard Statement on Standards for Attestation Engagements no. 16 (SSAE 16) method. HITECH-updated (Health Information Technology for Economic and Clinical Health Act) HIPAA (Health Insurance Portability and Accountability Act) rules are applied to ensure data protection and security of all customer data. The menstrual cycle and mood diary is recorded via the online platform Findmind (www.findmind.ch). Findmind treats data as confidential information, strives to ensure the privacy of the data and does not share information with third parties. It is operated exclusively on servers in Switzerland and stores all user data. For the dried blood spots (DBS), a sterile disposable lancet (Accu-Chek® Safe-T-Pro Plus) is used to collect up to five drops of blood (about 50 μL per drop), which are spotted onto standardized filter paper (No. 903 Whatman®, DBS Protein Saver Card). The samples are dried and subsequently stored in the laboratory freezer of the Institute of Psychology, University of Zurich at − 20 °C. DBS samples are analyzed in the biochemical laboratory of the Department of Clinical Psychology and Psychotherapy, University of Zurich, Switzerland (genetic and epigenetic analyses) and the Cytolab laboratory in Regensdorf, Switzerland (FSH and LH).

Saliva samples are collected with SaliCap sampling tubes of 2 mL capacity (IBL International GmbH, Hamburg, Germany). Participants are instructed to drool into the tube using a polypropylene straw (passive drool method). After collecting a full month of saliva samples, these can be returned directly to the Institute of Psychology during the study phase or stored at home in the freezer until the second laboratory appointment. The saliva samples are then thawed and centrifuged prior to biochemical analysis, using IBL Saliva Immunoassays (IBL International GmbH, Hamburg, Germany). All saliva samples are analyzed at the Cytolab laboratory in Regensdorf, Switzerland.

The Ava bracelet and its accompanying software is a CE-certified, Class 1 medical device approved by the Food and Drug Administration. It is worn at night on the wrist like a regular watch. The synchronization takes place every morning after waking up, with the corresponding application (Ava fertility tracker). The software runs on Apple and Android phones. The login for the Ava fertility tracker app corresponds to the subjects’ study ID number (personal code). Participants without a smartphone have been provided with an Android phone during the study phase by the University Research Priority Program Dynamics of Healthy Aging (funding source). The data recorded by the Ava bracelet are treated confidentially and in accordance with the privacy policy. Members of the Ava AG have signed a binding declaration of confidentiality. All data handling is subject to the data protection provisions approved by the Swiss Ethics Committee.

### Biochemical data analyses

Our biochemical laboratory at the Institute of Psychology, University of Zurich, performs the salivary and DBS analyses. As listed in Table 2, most endocrine parameters are assessed in saliva. Thawed saliva samples are centrifuged and analyzed using enzyme-linked immunosorbent assay (ELISA; IBL International GmbH, Hamburg, Germany). FSH and LH levels are determined in DBS samples (Table 2) using the MILLIPLEX MAP Human Pituitary Magnetic Bead Panel 1 (Merck, Darmstadt, Germany). Intra-assay variation for this kit for FSH and LH is less than 10% and inter-assay variation less than 15%. Sensitivity is 6.40 pg/mL for FSH and 1.34 pg/mL for LH. Sex hormone-binding globulin (SHBG) can be measured in DBS using Human SHBG ELISA Kit (DRG International, Marburg Germany) with intra- and inter-assay coefficients of variability of less than 10% [78]. In terms of immune markers, the Quantikine HS ELISA Human IL-6 (HS600B, R&D Systems, Minneapolis, MN) is recommended for analyzing interleukin 6 (IL- 6; 37) and the sandwich ELISA (BioCheck, Inc., Foster City, CA) for investigating C-reactive protein [79]. Intra- and inter-assay variation for these kits for IL-6 and CRP are less than 15%. Detection rates of these assays are 0.67 pg/ml for IL-6 and 3.00 mg/L for CRP.

The DNA extraction is assessed from DBS samples, as described in Table 2. The Qiagen QIAamp DNA Investigator Kit (Qiagen, Hombrechtikon, Switzerland) is used to extract genomic DNA from three (each 3 mm in diameter) punches of blood-soaked filter paper. These punches are then eluted in 30 μl of RNase-free water, and the DNA concentration is assessed using the Qubit Fluorometer (Thermo Fischer Scientific, Reinach, Switzerland). For epigenetic analyses, we perform a bisulfite conversion of DNA using the EZ 96-DNA Methylation-Gold Kit (Zymo Research, Luzern, Switzerland). The procedure for the next-generation sequencing (NGS) library preparation is described elsewhere by our research group [80].

### Statistical data analyses

A variety of statistical analytic strategies are planned for the different measures. Variables will be tested for normal distribution (Kolmogorov-Smirnov test) and homogeneity (Levene’s test). Skewed biological data will be logarithmically transformed [81]. Effect sizes will be estimated using eta-squared [82]. When investigating associations, correlation or partial correlation analyses will be conducted. If assumptions are violated, non-parametric methods will be applied. Regression analyses will be conducted to investigate the strength and form of associations between the various biopsychosocial markers and determinants and resilience- or depression-related factors. Analyses of variance, t-tests or Mann-Whitney U tests, depending on the level of measurement, are planned for the investigation of group differences between participants with and without a history of depression regarding different outcome measures associated with the menopausal transition. Furthermore, factor analyses will be used [81, 83] to examine which of the assessed variables related to resilience can be assigned to a common factor. For the longitudinal data evaluation of specific predictors of perimenopausal depression, multilevel analyses will be conducted [81]. In addition to inference methods, unsupervised methods (e.g. time series clustering methods [84, 85], machine learning algorithms [86]) will be necessary to analyze the massive volume of big data generated by the Ava bracelet.

The level of significance will be two-sided with *p* < .05, if not stated otherwise. The analysis will be run via the statistical software SPSS 25.0 (IBM, Armonk, NY, USA) and the open source software R (foundation for Statistical Computing, Vienna, Austria).

## Results

Of the 1121 women interested in participating in the Swiss Perimenopause Study, a total of 265 (23.64%) were found to be eligible for the study after applying the inclusion and exclusion criteria. Of these, 177 declared their consent to participate. Up until December 2019, 42 women (23.73% of the participants) were reported as dropouts. Excessive effort for study participation was stated as a major cause for dropping out. Hence, the total sample consisted of 135 perimenopausal women.

The sociodemographic data of the participants are shown in Table 4. The participants’ age at the point of study enrollment ranged from 40 to 56 years, with a mean of 48.60 years. Most of the women were Swiss (85.2%) and married (56.3%). With regard to highest educational attainment, 43.0% had completed university education and 37.0% had completed secondary school. In accordance with the STRAW criteria [76], a total of 43.7% were in the early phase of the menopausal transition, while 56.3% were in the late phase at the point of screening. Overall, 60.0% of the women showed no prior depression and 40.0% of the participants reported a history of depression.

**Table 4:** Baseline sample characteristics

	***N (%)***	***M (SD)***
Age		48.60 (3.87)
Nationality		
Swiss	115 (85.2%)	
German	17 (12.6%)	
Other	3 (2.1%)	
Marital status		
Single	34 (25.2%)	
Married	76 (56.3%)	
Registered partnership	4 (3%)	
Divorced	18 (13.3%)	
Widowed	3 (2.2%)	
Highest educational attainment		
Secondary school	50 (37.0%)	
Grammar school	27 (20.0%)	
University	58 (43.0%)	
Menopausal stage		
Early MT	59 (43.7%)	
Late MT	76 (56.3%)	
History of depression		
No	81 (60.0%)	
Yes	54 (40.0%)	

*Note. N* sample size, *M* mean, *SD* standard deviation, MT menopausal transition

## Discussion

The Swiss Perimenopause Study was established to investigate specific health-related markers associated with the menopausal transition. The aim of this large longitudinal study is to address the limited scientific understanding of the factors determining whether a woman undergoing the menopausal transition develops clinically relevant symptoms, such as those of a depressive disorder, or whether she manages to successfully adapt to the predominant biopsychosocial changes. Successful adaptation can be defined on a continuum of, for example, higher life satisfaction, lower psychological distress, better general psychological health, and milder menopausal complaints [14]. Thus, the study focuses on the perimenopause as the phase of strong hormonal fluctuations and the prevalence peak of burdensome symptoms.

Strengths of the Swiss Perimenopause Study include the longitudinal design and the in-depth investigation of the perimenopausal stage. Strict inclusion and exclusion criteria are deployed. All participants reported good to excellent subjective health at baseline. The gold-standard STRAW criteria were used to identify perimenopausal women on the basis of bleeding patterns. Individual menstrual cycle data are collected throughout the entire screening and study phase and matched with the assessment of mood changes. An extensive symptom assessment is carried out. Genetic and epigenetic analyses provide the opportunity to investigate inherited physical characteristics as well as gene-environment interactions. Repeated physiological data assessment enables the examination of patterns related to sleep disturbances and vasomotor symptoms. Furthermore, a comprehensive inspection of endocrine processes is planned, to complement the various psychosocial questionnaires applied in our study. Participants were randomly identified from the general population and represent an eligible sample for this longitudinal study on health-related factors of the menopausal transition.

There are a few limitations to consider: The study includes a predominantly well-educated sample, despite efforts to recruit women with different socioeconomic backgrounds. Additionally, the sample mostly consists of Caucasian women, although this corresponds to the distribution of the Swiss population. The initial goal to recruit a balanced sample size of women with and without a history of depression, with a sample size of 80 participants in each group, had to be discarded due to difficulties in enrolling eligible participants with prior depression. A total of 54 (40.0%) women with and 81 (60.0%) women without a history of depression were included in the final sample. Compared to the SWAN or the SMWHS studies, only a relatively small sample size and shorter study duration could be accomplished. However, a sufficiently large number of women has been achieved for all planned analyses, with adequate statistical power. Furthermore, the self-reported nature of all validated psychosocial questionnaires used might lead to a response bias and socially desirable answers. Thus, the evaluation of self-reported data using objective measures, where feasible, will be essential. Moreover, the strict inclusion and exclusion criteria applied in the Swiss Perimenopause Study offers the chance to investigate the actual changes and challenges associated with the perimenopause. However, as this is a very specific sample, the results cannot be applied to other subgroups of the general population such as younger women or postmenopausal women. Additionally, a selection bias has to be considered when interpreting the results. Women agreeing to participate in such a comprehensive examination about the menopausal transition might suffer more from menopausal symptoms, and women with a higher socioeconomic status might show a higher interest in participating in a scientific project.

There has been an increased scientific effort to address questions on the development of menopausal symptoms and depressed mood within the menopausal transition. Longitudinal data on specific factors associated with the menopausal transition are essential to foster our understanding of changes and symptoms experienced by middle-aged women. The Swiss Perimenopause Study, as one of the large studies investigating the menopausal transition, aims to contribute to increased scientific information about the biopsychosocial processes associated with this vulnerable time frame in a woman’s life and thus improve the clinical care of midlife women.

## Data Availability

Not available.

## Abbreviations

2D:4D: Second to Fourth Digit Ratio
ALSWH: Australian Longitudinal Study on Women’s Health
App: Application
BIA: Bioelectrical Impedance Analysis
BMI: Body Mass Index
BSI: Federal Office for Information Security (Bundesamt für Sicherheit in der Informationstechnik)
CAR: Cortisol Awakening Response
DBS: Dried Blood Spots
DSM: Diagnostic and Statistical Manual of Mental Disorders
ELISA: Enzyme-Linked Immunosorbent Assay
HIPAA: Health Insurance Portability and Accountability Act
HITECH: Health Information Technology for Economic and Clinical Health Act
HUNT: Nord-Trøndelag Health Study
IBL: Immuno Biological Laboratories
ISO: International Organization for Standardization
kHz: Kilohertz
LH: Luteinizing Hormone (LH)
MWMHP: Melbourne Women’s Midlife Health Project
MWHS: Massachusetts Women’s Health Study
NGS: Next-Generation Sequencing
POAS: Penn Ovarian Aging Study
SHBG: Sex hormone-binding globulin
SMWHS: Seattle Midlife Women’s Health Study
SSAE: Statement on Standards for Attestation Engagements
STRAW: Stages of Reproductive Aging Workshop
SWAN: Study of Women’s Health Across the Nation
